# Effect of storage on physicochemical attributes of ice cream enriched with microencapsulated anthocyanins from black carrot

**DOI:** 10.1002/fsn3.3384

**Published:** 2023-04-26

**Authors:** Aneela Shamshad, Iahtisham‐Ul‐Haq, Masood Sadiq Butt, Gulzar Ahmad Nayik, Sami Al Obaid, Mohammad Javed Ansari, Ioannis Konstantinos Karabagias, Nazmul Sarwar, Seema Ramniwas

**Affiliations:** ^1^ National Institute of Food Science and Technology Faculty of Food Nutrition and Home Sciences University of Agriculture Faisalabad Pakistan; ^2^ Kauser Abdulla Malik School of Life Sciences Forman Christian College (A Chartered University) Lahore Pakistan; ^3^ Department of Food Science and Technology Government Degree College Shopian Jammu and Kashmir India; ^4^ Department of Botany and Microbiology College of Science King Saud University Riyadh Saudi Arabia; ^5^ Department of Botany Hindu College Moradabad (Mahatma Jyotiba Phule Rohilkhand University Bareilly) Moradabad India; ^6^ Department of Food Science and Technology, School of Agricultural Sciences University of Patras Agrinio Greece; ^7^ Department of Food Processing and Engineering Chattogram Veterinary and Animal Sciences University Chattogram Bangladesh; ^8^ University Centre for Research and Development Chandigarh University Gharuan, Mohali India

**Keywords:** anthocyanin, black carrot, ice cream, microencapsulation, physiochemical

## Abstract

The present study was conducted to investigate the effect of storage on quality attributes of microencapsulated black carrot anthocyanins‐enriched ice cream. Purposely, black carrot anthocyanins were obtained using ethanolic extraction. Later on, extracts were acidified and microencapsulated with gum arabic and maltodextrin (1:1). Results showed that anthocyanin contents for T_3_ (9% microencapsulated anthocyanins powder‐enriched ice cream) had highest anthocyanin contents in the range of 143.21 ± 1.14 mg/100 g. However, during the storage, it was revealed that there was a slight decline in constituents concentration reasoned to oxygen exposure and interaction with other food ingredients. Similarly, for total phenolic content, the highest amount was found in T_3_ as 545.38 ± 4.34 mg GAE/100 g. The quality attributes of prepared ice cream treatments were also found acceptable till the end of the study (60 days). Conclusively, the addition of microencapsulated anthocyanins powder in ice cream proved to stabilize black carrot anthocyanins and contributed positively to the sensory characteristics of ice cream.

## Graphical abstract

The present research demonstrated the potential of gum arabic and maltodextrin for the production of microencapsulated anthocyanins powder for further utilization in ice cream. The resultant encapsulates showed significant potential to stabilize the anthocyanins during processing and storage of ice cream. Conclusively, the addition of microencapsulated anthocyanins powder in ice cream proved to stabilize black carrot anthocyanins and contributed positively to the sensory characteristics of ice cream.

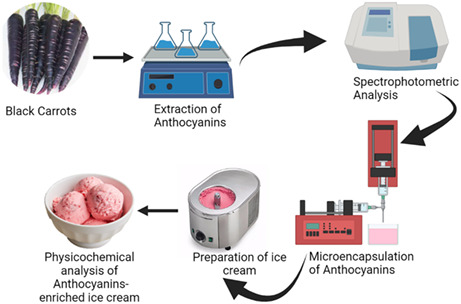

## 1 INTRODUCTION

In modern times, the demand for functional foods is increasing due to awareness regarding the benefits of bioactive ingredients, its physiological functions, and its positive influence on human health (Iahtisham‐Ul‐Haq, Butt, Shamshad, & Suleria, 2019). Functional foods referred to food as whole or food components are ingested to boost health and wellness. However, nutraceutical foods provide health as well as medical benefits including prevention and medication of various diseases (Onwulata, 2013).

Globally, use of functional and nutraceuticals as a therapy against various ailments is becoming popular. Another reason for using functional and nutraceutical foods is its low cost and comparative safety over medicines (Iahtisham‐Ul‐Haq, Butt, Randhawa, & Shahid, 2019b). These health‐boosting edibles have great importance because of its therapeutic and nutritional effects. Functional foods provide 30%–500% more benefits than conventional foods (Rajasekaran et al., 2008; Sakarkar & Deshmukh, 2011). Upcoming functional foods will have more capability to provide nutrients and health benefits but may be superior to supplements (Onwulata, 2013).

The carrot (*Daucus carota*), a member of family Umbelliferae, is a winter vegetable and exceedingly popular among vegetables. It is largely cultivated and consumed in Pakistan. Its cultivars are divided into two groups; carotene or western carrots (*Daucus carota* ssp. sativus var. sativus) and anthocyanin or eastern carrots (*Daucus carota* ssp. sativus var. atrorubens Alef). Although orange‐colored carrots are more consumed, recently there is a growing trend toward consumption of black carrots (*Daucus carota* L.) owing to its higher anthocyanin contents and astonishing antioxidant potential (Iahtisham‐Ul‐Haq, Butt, Shamshad, & Suleria, 2019). Currently, black carrots consumption is increasing in Western Europe, Middle Asia, Germany, Australia, and Turkey. In some countries like India and Pakistan, black carrots are rarely utilized as vegetables but a fermented beverage “kanji” is commonly used (Algarra et al., 2014; Khandare et al., 2011). Although black carrots also contain phenolic compounds and antioxidants like vitamins C and E, the main attractive feature of black carrots is its higher anthocyanin contents (Algarra et al., 2014; Iahtisham‐Ul‐Haq, Butt, Shamshad, & Suleria, 2019).

Anthocyanins are bioactive components of various fruits and vegetables and impart color to them. These have great importance in food quality because of its involvement in color and appearance. These impart purple, blue, and red colors to fruits, vegetables, and flowers. Anthocyanins are pivotal in nutraceuticals due to its additional health benefits. Because of the pharmacological properties of anthocyanin, these are utilized by humans for medicinal purposes. In addition to imparting color, anthocyanins also have a role in reducing risks of tumor, stroke, heart diseases, atherosclerosis, cancer, platelet aggregation, lipid peroxidation, inflammatory disorders, cognitive behavior, and diabetes (Khandare et al., 2011; Lee et al., 2005; Veigas et al., 2007). Apart from anthocyanins, betalains from beetroot have been used in the form of beverages to demonstrate its hepatic and renal health benefits that endorse the use of bioactive colorants in routine products for prevention from diseases (Iahtisham‐Ul‐Haq, Butt, Randhawa, & Shahid, 2019a, 2019b).

Microencapsulation is a technique to preserve the food material like food color or bioactive ingredients in a coating material forming nano‐ (<50 nm) or micro‐ (50 nm to 2 mm) particulates. It supplies the phytochemicals effectively, masks the undesirable flavor of core material, and avoids the destruction of bioactive components (Khan et al., 2022). Various microencapsulation techniques are widely applicable in food industries like freeze drying, spray drying, extrusion, liposome entrapment, emulsion, coacervation, and co‐crystallization (Ersus & Yurdagel, 2007; Khazaei et al., 2014; Saikia et al., 2015). Freeze drying is an expensive but simplest technique used for the encapsulation of natural aromas, water‐soluble extracts, and drugs (Khazaei et al., 2014). Anthocyanins are highly unstable during processing and storage. These are susceptible to various environmental factors like light, pH, temperature, and oxygen. As for freeze drying, it has no heat appliance, so this technique is effective in anthocyanins' stability. However, it is an expensive technique and applicable to the microencapsulation of pharmaceuticals and heat‐labile components (Kumar et al., 2022). Mostly, active packaging films are also being used for the preservation of the active components instead of its direct inclusion in the food system avoiding higher‐priced techniques (LakshmiBalasubramaniam et al., 2022).

Ice cream is a commonly consumed dairy product in the world and is enriched with valuable macro‐ and micronutrients like proteins, fats, carbohydrates, vitamins A, D, and E, and calcium. However, commercially available ice creams are deficient in natural antioxidants like natural colors, vitamin C, and anthocyanins. Enrichment of ice cream with anthocyanins will enhance its functional properties. Unfortunately, there is lack of vigorous technology regarding the application of such microcapsules in ice cream production (Çam et al., 2014). Recently, Kumar et al. (2022) have shared the perspective of using non‐dairy products for functional food development and enlightened us on the use of nutraceuticals in products.

Several types of ice creams are manufactured like plain ice cream, non‐fat, reduced fat, fruit mixed, and dry fruit ice creams. It also requires continuous mixing and incorporation of air, therefore deliberating the desired softness and pliability of ice cream (Kokini & van Aken, 2006). Ice cream production is remarkably high in the USA as it was 4.4 billion liters in 2010. Depending upon the requirement, 8%–10% of whole milk and 15% of processed milk are utilized in ice cream manufacturing (Goff & Hartel, 2013b). In this context, ice cream was selected as a carrier product to evaluate the behavior of black carrot anthocyanins encapsulating suitability and product acceptability.

## 2 MATERIALS AND METHODS

### 2.1 Procurement of raw materials

Black carrots were procured from a local market in Lahore, Pakistan. For the analysis, the reagents (analytical and HPLC grade) purchased from Merck (Merck KGaA, Darmstadt, Germany) were provided by a local supplier.

### 2.2 Preparation of raw material

Black carrots were washed and cleaned in order to remove adherent dust, dirt, and foreign materials in the Functional and Nutraceutical Food Research Section, National Institute of Food Science and Technology (NIFSAT), University of Agriculture, Faisalabad. Afterward, black carrots were stored at refrigeration temperature (4–6°C) until further use.

### 2.3 Preparation of black carrot extract

One hundred grams of black carrots were peeled and finely chopped. The sample was subjected to extraction with 100 mL EtOH:H_2_O (1:1, v/v), containing 0.01% HCl (37% v/v), and left with stirring at room temperature (25°C) for 2 h. Afterward, the liquid phase was separated and the extract was filtered through a 0.45 μm polytetrafluoroethylene (PTFE) membrane, followed by ethanol evaporation under vacuum. The extraction was carried out in triplicate.

### 2.4 Purification and concentration of anthocyanins

The resulting extract was purified by liquid–liquid extraction with a solvent mixture containing chloroform (50 mL), pentane (50 mL), and cyclohexane (50 mL), followed by concentration of aqueous phase via vacuum rotary evaporator at a temperature of 30°C and stored at 4°C in a dark place.

### 2.5 Microencapsulation of anthocyanins

Microencapsulation of anthocyanins was carried out using maltodextrin and gum arabic by freeze drying following the prescribed method of Khazaei et al. (2014). Wall materials including gum arabic and maltodextrin were mixed and dissolved in distilled water at ambient temperature (25 ± 1°C) to obtain 40% total solids concentration. The solution was kept in the refrigerator for complete hydration for 24 h. Then, the extract of anthocyanins from black carrots and wall materials were mixed in a weight ratio of 1:5 (extract:wall material). The pH of the mixtures was decreased to pH = 2 with HCl (1.5 N) to stabilize anthocyanins and then was mixed. The total anthocyanins of samples were measured through a differential pH method before drying. The solutions were dried in a freeze drier (Christ LCG Layo Chamber Guard 1,215,550 PMMA) for 42 h (−26 to −40°C, 5 mbar). Dried materials were ground using a pestle and mortar and passed through a 0.71 mm mesh and stored in brown glass bottles with screwed caps in a freezer (−0 to −10°C) until further use and analyses. The resultant powder was used in various concentrations in the manufacturing of ice cream (Table 1).

**TABLE 1:** Treatments used for microencapsulated black carrot anthocyanins powder‐enriched ice cream.

Treatments	Description
T_0_	Control
T_1_	3% microencapsulated anthocyanins‐enriched ice cream
T_2_	6% microencapsulated anthocyanins‐enriched ice cream
T_3_	9% microencapsulated anthocyanins‐enriched ice cream

### 2.6 Physicochemical analyses of ice cream

#### 2.6.1 pH

The pH of the ice cream was measured using a digital pH meter (InoLab 720, Germany). All measurements were done in triplicate. The pH meter was first calibrated using buffer solutions of pH 4, 7, and 10. The ice cream sample was taken into beaker and adjusted to room temperature. Electrode of pH meter was immersed in ice cream sample and reading was recorded after stabilization of pH meter reading.

#### 2.6.2 Titratable acidity

The acidity of samples was determined by titration method (AOAC, 2006). Acidity was determined by taking 10 g of ice cream sample in 100 mL Erlenmeyer flask, and after adding 2–3 drops of phenolphthalein, it was titrated against 0.1 N NaOH solution until pink color started to develop. The acidity was calculated by the following formula:

$$\text{Acidity}\%=\frac{0.009\times \text{volume used of}\;0.1N\;\text{NaOH}\;\left(\mathrm{mL}\right)}{\text{Weight of Sample}\;\left(g\right)}\times 100$$

#### 2.6.3 Overrun

The Overrun of ice cream was measured by a procedure outlined by El‐Samahy et al. (2009). The method was run in triplicate comparing the weight of a fixed volume of ice cream mix and ice cream. The results were expressed in %, defined as,

$$\text{Overrun}\;\left(\%\right)=\frac{M-I}{I}\times 100$$

where, M, weight of mix; I, weight of ice cream.

#### 2.6.4 Meltdown and specific weight

Meltdown enables an assessment to be made of the firmness of ice cream at the time of consumption. The rate of melting of all samples of ice cream was calculated according to the volume of liquid which drained off from 25 to 30 g of ice cream samples and placed on a sieve with 2 mm openings for 10 min at 25 ± 2°C (Santana et al., 2011). Similarly, the weight of a specific volume of the ice cream was noted and demonstrated as kg/gallon as a specific weight.

#### 2.6.5 Total anthocyanin content

The total anthocyanin content was determined by pH differential method proposed by Lee et al. (2005) using two buffer systems: potassium chloride buffer, pH 1 (0.025 M), and sodium acetate buffer, pH 4.5 (0.4 M). Samples were diluted in pH 1.0 and pH 4.5 buffers and absorbance measurements were made at 510 and 700 nm using 1 cm path length cuvettes. The pigment content was calculated and expressed as cyanidin 3‐glucoside (Cyd 3‐glu) per 100 g FW, using an extinction coefficient (e) of 26,900 L cm^−1^ mol^−1^ and a molar weight of 449.2 g mol^−1^.

$$\text{Absorbance}\;\left(A\right)=\left(A_{510\;\mathrm{nm}}–A_{700\;\mathrm{nm}}\right)\;\mathrm{pH}\;1.0–\left(A_{510\;\mathrm{nm}}–A_{700\;\mathrm{nm}}\right)\;\mathrm{pH}\;4.5.$$

Monomeric anthocyanin contents (mg L^−1^) were measured according to the following equation:

$$\mathrm{ACN}=\frac{A\times \mathrm{MW}\times \mathrm{DF}\times 1000}{e\times 1}$$

where, A, absorbance; MW, molar weight (449.2); DF, dilution factor; e, molar absorptivity (26,900).

#### 2.6.6 Total phenolic content

Total phenolic content (TPC) in black carrot extract was measured using the Folin–Ciocalteu method (Algarra et al., 2014), which is based on the reduction of phosphotungstic acid to phosphotungstic blue, and as a result, the absorbance is increased due to rise in number of aromatic phenolic groups. For this purpose, 50 μL of black carrot extract was separately added to test tube containing 250 μL of Folin–Ciocalteu reagent, 750 μL of 20% sodium carbonate solution, and the volume rose to 5 mL with distilled water. After 2 h, the absorbance was measured at 765 nm using a UV/Visible Light Spectrophotometer (IRMECO UV–Vis Model: U2020) against control that has all reaction reagents except the sample extract. Total phenolic content was expressed as gallic acid equivalents (mg gallic acid/100 g).

#### 2.6.7 Viscosity

The viscosity of the ice cream samples was measured after thawing at refrigeration temperature using viscometer Brookfield LVDVE‐230 (MA, USA) (Kaya & Tekin, 2001).

#### 2.6.8 Texture

The texture (firmness) of the prepared ice cream was measured by Texture Analyzer (TA‐HDi, Stable Microsystems, UK) using the protocol of Aime et al. (2001).

#### 2.6.9 Color

The products' surface color, L (lightness), a* (−a greenness; +a redness), and b* (−b blueness; +b yellowness), was measured according to the method of Ngadi et al. (2007).

### 2.7 Statistical analysis

All the data were subjected to analysis of variance (ANOVA) analysis using Statistix 8.1 software. The results were concluded significant at *p* < .05.

## 3 RESULTS AND DISCUSSION

Frozen food items are widely consumed across the world for its taste and convenience. Addition of bioactive ingredients and natural colors in such foods can result in the formulation of a better‐accepted product. The physicochemical analyses of microencapsulated anthocyanins powder‐enriched ice creams were carried out consisting of pH, acidity, overrun, meltdown, specific weight, anthocyanins, total phenolic content, viscosity, firmness, and color. In this respect, the results regarding each trait are discussed as follows:

### 3.1 pH

The effect of treatments and storage on pH of the microencapsulated anthocyanins‐enriched (MAE) ice cream was found significant. As evident from the average values (means) (Table 2), there was observed a clear decline in pH of ice creams due to treatments. It is inferred that a maximum value of pH was observed in T0 (control) as 6.67 ± 0.01, followed by T1 (3% microencapsulated anthocyanins powder‐enriched ice cream) and T2 (6% microencapsulated anthocyanins powder‐enriched ice cream) (i.e., 6.55 ± 0.02 and 6.43 ± 0.01, respectively). However, the minimum value for the attribute was observed in T3 (9% microencapsulated anthocyanins powder‐enriched ice cream) as 6.32 ± 0.02. The possible reason for the decrease in pH among treatments appears to be the enrichment of ice cream with microencapsulated anthocyanins powder. Over the storage, there was observed a momentous decrease in the pH of ice cream which ranged between 6.53 ± 0.02 and 6.47 ± 0.01 from initiation to the termination of the present research trial. The recorded values on the 15th, 30th, and 45th days of storage were 6.51 ± 0.01, 6.49 ± 0.01, and 6.48 ± 0.01, respectively. The decrease in pH over the storage may be due to the conversion of sugar or starch molecules into acids. Among the treatments, the maximum decline in acidity was observed in T3 as 6.38 ± 0.03 at day 0 to 6.29 ± 0.02 after 60 days of storage, followed by T0 and T2 ranging from 6.70 ± 0.01 and 6.45 ± 0.02 to 6.64 ± 0.01, and 6.40 ± 0.01 from initiation to the termination day, respectively. However, the minimum drop in pH was recorded in T1 as 6.57 ± 0.02 to 6.53 ± 0.02 from 0 to 60 days of storage.

**TABLE 2:** Means for pH of microencapsulated anthocyanins powder‐enriched ice creams.

Storage (days)	Treatments				Means
	T_0_	T_1_	T_2_	T_3_	
0	6.70 ± 0.01	6.57 ± 0.02	6.45 ± 0.02	6.38 ± 0.03	6.53 ± 0.02^a^
15	6.68 ± 0.01	6.57 ± 0.02	6.45 ± 0.01	6.34 ± 0.01	6.51 ± 0.01^a^
30	6.67 ± 0.01	6.55 ± 0.02	6.44 ± 0.01	6.31 ± 0.01	6.49 ± 0.01^b^
45	6.65 ± 0.01	6.54 ± 0.02	6.42 ± 0.02	6.30 ± 0.01	6.48 ± 0.01^b^
60	6.64 ± 0.01	6.53 ± 0.02	6.40 ± 0.01	6.29 ± 0.02	6.47 ± 0.01^c^
Means	6.67 ± 0.01^a^	6.55 ± 0.02^b^	6.43 ± 0.01^c^	6.32 ± 0.02^d^	

*Note*: Means carrying the same letters are statistically non‐significant (*p* ≥ .05).

T_0_ = Control.

T_1_ = 3% microencapsulated anthocyanins powder‐enriched ice cream.

T_2_ = 6% microencapsulated anthocyanins powder‐enriched ice cream.

T_3_ = 9% microencapsulated anthocyanins powder‐enriched ice cream.

pH has a direct influence on flavor perception in dairy products. According to Homayouni et al. (2008), normal pH of ice cream is about 6–7. The outcomes of current research are in conformity with the earlier work of Murtaza et al. (2004), who reported that there was a gradual decrease in pH (6.85–6.78) throughout the storage period. The reason for the decrease in pH values was the addition of different stabilizers/emulsifiers and conversion of lactose into lactic acid during storage due to lactic acid bacteria. The findings of the instant research are also in harmony with the earlier conclusions of Bajwa et al. (2003). The authors used different concentrations of strawberry pulp to investigate its effects on ice cream properties. They confirmed a decreasing trend in pH (6.26–6.09) due to the presence of ascorbic acid in strawberries.

### 3.2 Titratable acidity (%)

Table 3 exhibits the mean values for titratable acidity of microencapsulated anthocyanins powder‐enriched ice cream. It is obvious from the table that there is a clear difference in mean values of titratable acidity of ice creams along the treatments. It is inferred that a maximum value of titratable acidity was observed in T_3_ (9% microencapsulated anthocyanins powder‐enriched ice cream) as 0.20 ± 0.01%, followed by T_2_ (6% microencapsulated anthocyanins powder‐enriched ice cream) and T_1_ (3% microencapsulated anthocyanins powder‐enriched ice cream) as 0.18 ± 0.01 and 0.16 ± 0.01%, respectively. However, the minimum value for the attribute was observed in T_0_ (control) as 0.15 ± 0.01%. An increase in acidity among treatments may be due to the addition of microencapsulated anthocyanins powder in ice creams. Over the storage, there was observed a non‐momentous increase in titratable acidity of ice creams which ranged between 0.17 ± 0.01 and 0.18 ± 0.01% from initiation to the termination of the research trial. The recorded values on the 15th and 45th days of storage were also 0.17 ± 0.01 and 0.17 ± 0.01%, respectively. The possible reason behind the increase in acidity during storage is the decline in pH. Among the treatments, the maximum decline in titratable acidity was observed in T_0_, T_1_, and T_3_ ranging from 0.15 ± 0.01, 0.16 ± 0.02, and 0.19 ± 0.01 at day 0, to 0.16 ± 0.01, 0.17 ± 0.01, and 0.2 ± 0.01% after 60 days of storage. However, the minimum drop off in titratable acidity was recorded in T_2_ as 0.18 ± 0.01 to 0.18 ± 0.0% from 0 to 60 days of storage.

**TABLE 3:** Means for titratable acidity (%) of microencapsulated anthocyanins powder‐enriched ice creams.

Storage (days)	Treatments				Means
	T_0_	T_1_	T_2_	T_3_	
0	0.15 ± 0.01	0.16 ± 0.02	0.18 ± 0.01	0.19 ± 0.01	0.17 ± 0.01
15	0.15 ± 0.02	0.16 ± 0.01	0.18 ± 0.01	0.19 ± 0.01	0.17 ± 0.01
30	0.15 ± 0.01	0.16 ± 0.01	0.18 ± 0.01	0.19 ± 0.01	0.17 ± 0.01
45	0.15 ± 0.01	0.16 ± 0.01	0.18 ± 0.01	0.20 ± 0.01	0.17 ± 0.01
60	0.16 ± 0.01	0.17 ± 0.01	0.18 ± 0.01	0.20 ± 0.01	0.18 ± 0.01
Means	0.15 ± 0.01^c^	0.16 ± 0.01^bc^	0.18 ± 0.01^b^	0.20 ± 0.01^a^	

*Note*: Means carrying the same letters are statistically non‐significant (*p* ≥ .05).

T_0_ = Control.

T_1_ = 3% microencapsulated anthocyanins powder‐enriched ice cream.

T_2_ = 6% microencapsulated anthocyanins powder‐enriched ice cream.

T_3_ = 9% microencapsulated anthocyanins powder‐enriched ice cream.

Acidity is the measure of the amount of acids in any food sample. Ideal acidity of ice cream is 0.198% (Bhandari, 2001). The findings of the current investigation are in accordance with the earlier work of Bajwa et al. (2003). They checked the effect of different concentrations of strawberry pulp on ice cream properties. They deduced from their investigations that there occurs an increase in titratable acidity over the storage due to the presence of ascorbic acid in strawberries. The outcomes of instant research are also in harmony with the research work of Murtaza et al. (2004), who confirmed the same increasing pattern in titratable acidity of ice cream throughout the storage period. The reason for such an increase was the addition of different stabilizer/emulsifiers and conversion of lactose into lactic acid due to lactic acid bacteria.

### 3.3 Overrun

The mean values for overrun (%) of enriched ice creams are exhibited in Table 4. It is palpable from mean values that the maximum value was recorded for T_3_ (9% microencapsulated anthocyanins powder‐enriched ice cream) as 53.37 ± 1.60%, followed by T_2_ (6% microencapsulated anthocyanins powder‐enriched ice cream) and T_1_ (3% microencapsulated anthocyanins powder‐enriched ice cream), that is, 50.72 ± 3.04 and 47.17 ± 1.89%, respectively. However, the minimum value for the trait was recorded in T_0_ (control) as 44.34 ± 2.22%.

**TABLE 4:** Means for overrun (%), meltdown (%), and specific weight (kg/gallon) of microencapsulated anthocyanins powder‐enriched ice creams.

Treatments	Overrun (%)	Meltdown (%)	Specific weight (kg/gallon)
T_0_	44.34 ± 2.22^c^	29.19 ± 1.75^a^	2.62 ± 0.04^a^
T_1_	47.17 ± 1.89^b^	27.50 ± 1.10^ab^	2.57 ± 0.03^ab^
T_2_	50.72 ± 3.04^ab^	25.62 ± 1.28^b^	2.51 ± 0.05^b^
T_3_	53.37 ± 1.60^a^	22.53 ± 0.68^c^	2.46 ± 0.03^c^

*Note*: Means carrying the same letters are statistically non‐significant (*p* ≥ .05).

T_0_ = Control.

T_1_ = 3% microencapsulated anthocyanins powder‐enriched ice cream.

T_2_ = 6% microencapsulated anthocyanins powder‐enriched ice cream.

T_3_ = 9% microencapsulated anthocyanins powder‐enriched ice cream.

Increase in the volume of the ice cream with respect to the volume of the initial mix, incorporating a certain amount of air into mix, and expansion due to freezing is called overrun. Overrun is largely influenced by stabilizer, emulsifier, fat content, and processing conditions (Chang & Hartel, 2002). Overrun of ice cream must fall within the range 60%–100%. By volume 40%–50% of air is whipped into a typical ice cream (Roland et al., 1999). The findings of the present study are in general agreement with the results reported by Murtaza et al. (2004) who evaluated the effect of different stabilizers/emulsifiers on ice cream and reported similar trends.

### 3.4 Meltdown

Tremendous variations took place in ice cream due to the addition of various concentrations of microencapsulated anthocyanins powder. The mean values for meltdown (%) of enriched ice creams are exhibited in Table 4. It is obvious from the mean values that the maximum value was recorded for T_0_ (control) as 29.19 ± 1.75%, followed by T_1_ (3% microencapsulated anthocyanins powder‐enriched ice cream) and T_2_ (6% microencapsulated anthocyanins powder‐enriched ice cream), that is, 27.50 ± 1.10 and 25.62 ± 1.28%, respectively. However, the minimum value for the trait was recorded in T_3_ (9% microencapsulated anthocyanins powder‐enriched ice cream) as 22.53 ± 0.68%.

Meltdown is one of the important properties of frozen dairy products. It is influenced by various factors including the amount of air incorporated, ice crystals, and networking of fat globules (Muse & Hartel, 2004). Good‐quality ice creams have a lower meltdown while fast‐melted ice cream is less resistant to heat shock. A lower melting rate in ice cream with a higher overrun is mainly attributed to reduced heat transfer across air bubbles (Alizadeh et al., 2014). Low freezing point is the primary cause of rapid melting (Goff, 2002; Goff & Hartel, 2013a). The melting of ice cream is controlled by the outside temperature and heat transfer. Homogenization improves the meltdown properties of ice cream (Goff, 2002). Ice cream has desirable melting quality when melted ice cream is very similar in characteristics to that of the original ice cream mix (Bhandari, 2001).

### 3.5 Specific weight

The mean values for the specific weight (kg/gallon) of enriched ice creams are exhibited in Table 4. It is palpable from the mean values that the maximum value was recorded for T_0_ (control) as 2.62 ± 0.04 kg/gallon, followed by T_1_ (3% microencapsulated anthocyanins powder‐enriched ice cream) and T_2_ (6% microencapsulated anthocyanins powder‐enriched ice cream); that is, 2.57 ± 0.03 and 2.51 ± 0.05 kg/gallon, respectively. However, the minimum value for the trait was recorded in T_3_ (9% microencapsulated anthocyanins powder‐enriched ice cream) as 2.46 ± 0.03 kg/gallon.

The findings of the present study research are in general agreement with the earlier work of El‐Samahy et al. (2009), who evaluated the effect of different concentrations of cactus pear pulp in ice cream. They reported a value of 3.25 ± 0.30 kg/gallon and 3.91 ± 0.3 kg/gallon in control and treated samples, respectively.

### 3.6 Anthocyanin contents

For microencapsulated anthocyanins powder‐enriched ice cream, the means regarding anthocyanin contents are given in Table 5. From the means, the minimum value for the trait was recorded for T_1_ (3% microencapsulated anthocyanins powder‐enriched ice cream) as 43.16 ± 1.17 mg/100 g, followed by T_2_ (6% microencapsulated anthocyanins powder‐enriched ice cream) as 92.63 ± 1.17 mg/100 g. However, the maximum value for the TPC was observed in T_3_ (9% microencapsulated anthocyanins powder‐enriched ice cream) as 143.21 ± 1.14 mg/100 g. The inclining trend in the case of anthocyanins in T_1_, T_2_, and T_3_ is probably related to the inclusion of microencapsulated anthocyanins powder into the ice creams. Over the storage, it is evident from the table that the storage was affected differently with regards to anthocyanin contents of ice cream. Values regarding this trait were 69.81 ± 0.85 mg/100 g at 0 day while a gradual increase was noted on the 15th days of storage as 72.3 ± 1.09 mg/100 g. However, a clear decline was observed on 45th and 60th days of storage (i.e., 68.6 ± 0.85 and 67.18 ± 0.8 mg/100 g, respectively). A possible reason behind the decline in storage may be due to the exposure to oxygen and interaction with other food ingredients. Among the treatments, the same trend was recorded for the trait, that is, 143.64 ± 1.05, 92.28 ± 1.16, and 43.31 ± 1.21 mg/100 g at the initiation of the research trial and 146.98 ± 1.64, 95.43 ± 1.24, and 46.8 ± 1.47 mg/100 g at 15th day in T_3_, T_2_, and T_1_, respectively, while at the 60th day of storage, the values were 139.57 ± 1.05, 89.64 ± 0.96, and 39.53 ± 1.17 mg/100 g in T_3_, T_2_, and T_1_, respectively. However, no anthocyanins were recorded in T_0_ as evident from Table 5.

**TABLE 5:** Means for anthocyanins contents (mg/100 g) of microencapsulated anthocyanins powder‐enriched ice creams.

Storage (days)	Treatments				Means
	T_0_	T_1_	T_2_	T_3_	
0		43.31 ± 1.21	92.28 ± 1.16	143.64 ± 1.05	69.81 ± 0.85^c^
15		46.80 ± 1.47	95.43 ± 1.24	146.98 ± 1.64	72.30 ± 1.09^a^
30	ND	44.57 ± 0.91	94.37 ± 1.20	144.49 ± 0.94	70.86 ± 0.76^b^
45		41.60 ± 1.08	91.42 ± 1.31	141.36 ± 1.01	68.60 ± 0.85^d^
60		39.53 ± 1.17	89.64 ± 0.96	139.57 ± 1.05	67.18 ± 0.80^e^
Means		43.16 ± 1.17^c^	92.63 ± 1.17^b^	143.21 ± 1.14^a^	

*Note*: Means carrying the same letters are statistically non‐significant (*p* ≥ .05).

T_0_ = Control.

T_1_ = 3% microencapsulated anthocyanins powder‐enriched ice cream.

T_2_ = 6% microencapsulated anthocyanins powder‐enriched ice cream.

T_3_ = 9% microencapsulated anthocyanins powder‐enriched ice cream.

Abbreviations: ND, not detected.

The findings of the current study are in general accordance with the earlier work of Mazur et al. (2014) who checked the effect of storage on color, anthocyanin, and ascorbic acid contents of raspberry jam. There occurred a 58% decline in anthocyanin content of raspberry jam during 3 months of storage. They also reported that storage had a significant effect on ascorbic contents of raspberry jam. The research outcomes are also in concordance with the earlier work of Rababah et al. (2011) who reported a significant decrease in anthocyanin content of fig and apricot jams during 5 months of storage.

### 3.7 Total phenolic content

From the mean values, it can be inferred that there were significant variations in total phenolic content of the treatments as shown in Table 6. The highest amount of total phenolics was found to be in the T_3_ (9% microencapsulated anthocyanins powder‐enriched ice cream) as depicted by the mean value, that is, 545.38 ± 4.34 mg GAE/100 g, followed by T_2_ (6% microencapsulated anthocyanins powder‐enriched ice cream) as 352.76 ± 4.47 mg GAE/100 g. Likewise, for T_1_ (3% microencapsulated anthocyanins powder‐enriched ice cream), the mean value was 164.38 ± 4.45 mg GAE/100 g. However, there was no measured total phenolic content in T_0_ (control). The incline in TPC of T_1_, T_2_, and T_3_ seems to be due to the elevated levels of anthocyanins in enriched ice creams. Regarding the storage, it is evident from the table that the storage affected differently the total phenolic content of ice creams. A significant increase was recorded on the 15th day of storage; that is, the values noted on day 0 were 265.85 ± 3.24 mg GAE/100 g, while 275.35 ± 4.15 mg GAE/100 g on 15th day. However, a gradual decline in PPC was observed on 60th day of storage equal to 255.86 ± 3.04 mg GAE/100 g. Moreover, a reason for the decline in TPC over storage could possibly be due to the decrease in anthocyanin contents owed to environmental factors. Among the treatments, the same trend was observed in T_1_, T_2_, and T_3_ as 164.95 ± 4.6, 351.43 ± 4.4, and 547.02 ± 3.98 mg GAE/100 g at the initiation of the research trial, while 178.24 ± 5.61, 363.43 ± 4.72, and 559.74 ± 6.26 mg GAE/100 g were the TPC values on the 15th day of storage and 150.55 ± 4.47, 341.38 ± 3.67, and 531.52 ± 4.01 mg GAE/100 g at the termination of the study. Moreover, there were no measured anthocyanins content in T_0_ at any time during storage.

**TABLE 6:** Means for total phenolic contents (mg GAE/100 g) of microencapsulated anthocyanins powder‐enriched ice creams.

Storage (days)	Treatments				Means
	T_0_	T_1_	T_2_	T_3_	
0		164.95 ± 4.60	351.43 ± 4.40	547.02 ± 3.98	265.85 ± 3.24^c^
15		178.24 ± 5.61	363.43 ± 4.72	559.74 ± 6.26	275.35 ± 4.15^a^
30	ND	169.74 ± 3.45	359.38 ± 4.55	550.27 ± 3.58	269.85 ± 3.23^b^
45		158.44 ± 4.11	348.16 ± 5.01	538.34 ± 3.85	261.24 ± 3.24^d^
60		150.55 ± 4.47	341.38 ± 3.67	531.52 ± 4.01	255.86 ± 3.04^e^
Means		164.38 ± 4.45^c^	352.76 ± 4.47^b^	545.38 ± 4.34^a^	

*Note*: Means carrying the same letters are statistically non‐significant (*p* ≥ .05).

T_0_ = Control.

T_1_ = 3% microencapsulated anthocyanins powder‐enriched ice cream.

T_2_ = 6% microencapsulated anthocyanins powder‐enriched ice cream.

T_3_ = 9% microencapsulated anthocyanins powder‐enriched ice cream.

Abbreviations: ND, not detected.

The results of the present study are in line with those of Rababah et al. (2011) who reported a significant decrease in the total phenolic content of fig and apricot jams during 5 months of storage. The findings of the instant research are also in agreement with the earlier conclusions of Patras et al. (2011) who studied the effect of time and temperature on bioactive compounds of strawberry jam and concluded a momentous decline (24.2%–31.6%) in total phenolics during 28 days of storage.

### 3.8 Viscosity

From the average values shown in Table 7, it is explicated that there was a gradual improvement in viscosity of microencapsulated anthocyanins powder‐enriched ice creams. It is therefore clear that the maximum value was documented for T_3_ (9% microencapsulated anthocyanins powder‐enriched ice cream) as 75.81 ± 2.253 cP, followed by T_2_ (6% microencapsulated anthocyanins powder‐enriched ice cream) as 68.88 ± 2.008 cP. Likewise, the average value for the trait was observed as 63.81 ± 1.715 cP for T_1_ (3% microencapsulated anthocyanins powder‐enriched ice cream). However, the minimum value for the attribute was recorded in T_0_ (control) as 58.76 ± 4.072 cP. The increase in viscosity for T_1_, T_2_, and T_3_ is attributed to the addition of microencapsulated anthocyanins powder in ice creams. Over the storage, there was observed a momentous decrease in viscosity of ice cream which ranged between 82.68 ± 2.784 and 52.03 ± 2.074 cP from initiation to the termination of the research trial. The recorded values on the 15th, 30th, and 45th days of storage were 74.16 ± 2.414, 66.26 ± 3.112, and 58.95 ± 2.176 cP, respectively. However, the storage decrease in viscosity may be due to the decrease in firmness. Among the treatments, the maximum decline in viscosity was observed in T_0_ and T_1_ ranging from 75.73 ± 6.985 and 80.23 ± 1.014 cP on day 0 to 43.16 ± 2.296 and 48.03 ± 1.814 cP after 60th day of storage, followed by T_2_ as 84.42 ± 0.586 to 54.31 ± 2.44 cP from initiation to termination day. However, the minimum drop in viscosity was recorded in T_3_ as 90.35 ± 2.55 to 62.60 ± 1.745 cP from day 0 to day 60 of storage.

**TABLE 7:** Means for viscosity (cP) of microencapsulated anthocyanins powder‐enriched ice creams.

Storage (days)	Treatments				Means
	T_0_	T_1_	T_2_	T_3_	
0	75.73 ± 6.99	80.23 ± 1.01	84.42 ± 0.59	90.35 ± 2.55	82.68 ± 2.78^a^
15	66.31 ± 2.79	72.01 ± 2.30	75.97 ± 2.61	82.36 ± 1.96	74.16 ± 2.41^ab^
30	57.85 ± 4.86	63.55 ± 2.45	68.38 ± 2.95	75.26 ± 2.19	66.26 ± 3.11^b^
45	50.75 ± 3.43	55.23 ± 0.99	61.34 ± 1.46	68.47 ± 2.82	58.95 ± 2.18^bc^
60	43.16 ± 2.30	48.03 ± 1.81	54.31 ± 2.44	62.60 ± 1.75	52.03 ± 2.07^c^
Means	58.76 ± 4.07^d^	63.81 ± 1.71^c^	68.88 ± 2.01^b^	75.81 ± 2.25^a^	

*Note*: Means carrying the same letters are statistically non‐significant (*p* ≥ .05).

T_0_ = Control.

T_1_ = 3% microencapsulated anthocyanins powder‐enriched ice cream.

T_2_ = 6% microencapsulated anthocyanins powder‐enriched ice cream.

T_3_ = 9% microencapsulated anthocyanins powder‐enriched ice cream.

Viscosity is a factor to measure resistance to flow usually in centipoise (cP). The factors affecting viscosity of ice cream are temperature, fat globule size, type, and amount of stabilizer. It also contributes to the mouthfeel and flavor of ice cream (Hematyar et al., 2012). The findings of the current research are in accordance with an earlier investigation by Butt et al. (1999) who elucidated the effect of different stabilizers/emulsifiers on ice cream. They reported that there was an obvious decline in the viscosity of ice cream due to the addition of various stabilizers/emulsifiers during storage.

### 3.9 Firmness

The mean values of firmness of enriched ice creams are exhibited in Table 8. Among the treatments, significant variations with respect to firmness were noted. In this regard, a maximum value for this parameter was observed for T_3_ (9% microencapsulated anthocyanins powder‐enriched ice cream) as 4.11 ± 0.058 N, followed by T_2_ (6% microencapsulated anthocyanins powder‐enriched ice cream) and T_1_ (3% microencapsulated anthocyanins powder‐enriched ice cream) as 3.81 ± 0.061 and 3.49 ± 0.047 N. However, a minimum value for firmness was recorded in T_0_ (Control) as 3.08 ± 0.017 N. Increment in firmness of T_1_, T_2_, and T_3_ is attributed to the stabilizing effect of the enrichment of ice creams with microencapsulated anthocyanins powder that contains maltodextrin and gum arabic as wall materials. Over the storage, a steady decline in firmness was observed ranging from 3.72 ± 0.047 N on day 0 to 3.53 ± 0.045 N after 60 days of storage. The possible reason for this trend in firmness is due to the retrogradation of starch molecules and dextrin degradation. Similarly, a clear demotion in firmness values in T_3_ was also noticed ranging from 4.22 ± .06 to 4 ± 0.056 N on day 0 and day 60 of storage, respectively. Minimum values for firmness were recorded in T_0_ from 3.15 ± 0.018 to 2.99 ± 0.017 N.

**TABLE 8:** Means for firmness (N) of microencapsulated anthocyanins powder‐enriched ice creams.

Storage (days)	Treatments				Means
	T_0_	T_1_	T_2_	T_3_	
0	3.15 ± 0.02	3.58 ± 0.05	3.91 ± 0.06	4.22 ± 0.06	3.72 ± 0.05^a^
15	3.12 ± 0.02	3.53 ± 0.05	3.85 ± 0.06	4.17 ± 0.06	3.67 ± 0.05^b^
30	3.09 ± 0.02	3.51 ± 0.05	3.83 ± 0.06	4.13 ± 0.06	3.64 ± 0.05^b^
45	3.04 ± 0.02	3.45 ± 0.05	3.77 ± 0.06	4.06 ± 0.06	3.58 ± 0.05^c^
60	2.99 ± 0.02	3.4 ± 0.05	3.71 ± 0.06	4.00 ± 0.06	3.53 ± 0.04^d^
Means	3.08 ± 0.02^d^	3.49 ± 0.05^c^	3.81 ± 0.06^b^	4.11 ± 0.06^a^	

*Note*: Means carrying the same letters are statistically non‐significant (*p* ≥ .05).

T_0_ = Control.

T_1_ = 3% microencapsulated anthocyanins powder‐enriched ice cream.

T_2_ = 6% microencapsulated anthocyanins powder‐enriched ice cream.

T_3_ = 9% microencapsulated anthocyanins powder‐enriched ice cream.

The firmness of ice cream is measured as the resistance of ice cream to deformation when an external force is applied. Both the ice crystal size and ice phase volume contribute to the firmness of ice cream. This factor may be used as a measure of ice crystal growth. Ice cream resistance to mechanical force provided by the tongue, teeth, and upper palate will give the overall perception of firmness (Muse & Hartel, 2004).

### 3.10 Color indices

The color is a primary aspect of the product for its acceptance or rejection by the consumers. In this regard, the CIELAB parameters of the microencapsulated black carrot anthocyanins‐enriched ice creams were analyzed and illustrated in Table 9.

**TABLE 9:** Means for color indices of microencapsulated anthocyanins powder‐enriched ice creams.

Color indices	Storage (days)	Treatments				Means
		T_0_	T_1_	T_2_	T_3_	
L	0	95.90 ± 1.68	53.06 ± 1.08	45.08 ± 1.07	46.07 ± 0.97	60.03 ± 1.20^a^
	15	96.50 ± 1.45	52.60 ± 0.91	44.32 ± 1.06	45.62 ± 1.51	59.76 ± 1.23^ab^
	30	95.85 ± 1.12	53.13 ± 1.06	44.88 ± 0.98	45.99 ± 1.53	59.96 ± 1.17 ^ab^
	45	95.07 ± 1.64	52.43 ± 0.64	43.72 ± 1.33	44.62 ± 1.09	58.96 ± 1.18^b^
	60	94.61 ± 0.67	51.76 ± 1.54	42.23 ± 1.98	43.63 ± 1.58	58.06 ± 1.44^b^
	Means	95.59 ± 1.31^a^	52.6 ± 1.05^b^	44.05 ± 1.28^c^	45.19 ± 1.34^c^	
a*	0	−1.04 ± 0.44	29.21 ± 1.14	30.04 ± 0.96	33.18 ± 1.04	22.85 ± 0.89^a^
	15	−1.58 ± 0.10	28.78 ± 1.02	29.75 ± 1.09	32.65 ± 1.00	22.40 ± 0.80^ab^
	30	−2.07 ± 0.45	28.04 ± 1.41	29.21 ± 1.13	32.14 ± 1.57	21.83 ± 1.14^b^
	45	−2.35 ± 0.53	27.75 ± 0.75	28.96 ± 1.61	31.79 ± 1.46	21.54 ± 1.09^bc^
	60	−2.78 ± 0.70	27.15 ± 1.08	28.31 ± 1.23	31.19 ± 0.92	20.97 ± 0.98^c^
	Means	−1.97 ± 0.45^d^	28.19 ± 1.08^c^	29.25 ± 1.2^b^	32.19 ± 1.2^a^	
b*	0	7.16 ± 1.09	5.14 ± 0.97	9.08 ± 0.95	11.05 ± 0.96	8.11 ± 0.99^a^
	15	6.89 ± 0.81	4.82 ± 1.36	8.74 ± 0.89	10.75 ± 0.92	7.80 ± 1.0a^b^
	30	6.48 ± 0.94	4.46 ± 1.32	8.39 ± 1.00	10.31 ± 1.05	7.41 ± 1.08^a‐c^
	45	6.14 ± 1.08	4.08 ± 1.15	7.99 ± 1.26	9.97 ± 0.80	7.04 ± 1.07^bc^
	60	5.90 ± 1.27	3.81 ± 1.50	7.67 ± 1.12	9.46 ± 0.89	6.71 ± 1.20^c^
	Means	6.51 ± 1.04^c^	4.46 ± 1.26^d^	8.37 ± 1.04^b^	10.31 ± 0.92^a^	
Chroma	0	7.24 ± 1.13	29.67 ± 1.06	31.39 ± 1.07	34.97 ± 1.29	25.82 ± 1.14^a^
	15	7.07 ± 0.77	29.20 ± 1.23	31.02 ± 0.92	34.39 ± 0.68	25.42 ± 0.9^ab^
	30	6.81 ± 0.93	28.42 ± 1.19	30.40 ± 1.25	33.75 ± 1.81	24.84 ± 1.30^bc^
	45	6.59 ± 1.06	28.06 ± 0.90	30.07 ± 1.26	33.32 ± 1.59	24.51 ± 1.20^bc^
	60	6.53 ± 1.40	27.44 ± 1.15	29.35 ± 1.04	32.60 ± 0.81	23.98 ± 1.10^c^
	Means	6.85 ± 1.06^d^	28.56 ± 1.11^c^	30.44 ± 1.11^b^	33.81 ± 1.24^a^	
Hue angle	0	98.09 ± 2.82^d^	10.01 ± 2.01^f^	16.81 ± 1.49^e^	18.4 ± 0.97^e^	35.83 ± 1.82^c^
	15	103.09 ± 2.35^c^	9.36 ± 2.36^f^	16.39 ± 1.99^e^	18.25 ± 1.95^e^	36.77 ± 2.16^bc^
	30	107.82 ± 3.78^b^	9.12 ± 3.1.0^f^	16.01 ± 1.61^e^	17.75 ± 0.92^e^	37.68 ± 2.35^b^
	45	111.17 ± 4.78^ab^	8.31 ± 2.11^f^	15.5 ± 3.07^e^	17.41 ± 0.85^e^	38.10 ± 2.70^a^
	60	115.31 ± 3.57^a^	7.96 ± 3.01^f^	15.19 ± 2.55^e^	16.89 ± 1.76^e^	38.84 ± 2.72^a^
	Means	107.10 ± 3.46^a^	8.95 ± 2.52^c^	15.98 ± 2.14^b^	17.74 ± 1.29^b^	

*Note*: Means carrying the same letters are statistically non‐significant (*p* ≥ .05).

T_0_ = Control.

T_1_ = 3% microencapsulated anthocyanins powder‐enriched ice cream.

T_2_ = 6% microencapsulated anthocyanins powder‐enriched ice cream.

T_3_ = 9% microencapsulated anthocyanins powder‐enriched ice cream.

#### 3.10.1 L* value

It can be inferred that the treatments behaved differently with regards to L* value of the ice creams. The maximum value was observed in T_0_ (control) as evident from the mean (95.59 ± 1.31). Among the treatments, the decrease in L* values in T_1_ (3% microencapsulated anthocyanins powder‐enriched ice cream) and T_2_ (6% microencapsulated anthocyanins powder‐enriched ice cream) was monitored as 52.60 ± 1.05 and 44.05 ± 1.28, respectively. However, a minor increase was observed in T_3_ (9% microencapsulated anthocyanins powder‐enriched ice cream) at 45.19 ± 1.34. Over the storage, there were noted significant differences in the mean values of the L color parameter, there was a decrease in L* values as 60.03 ± 1.20 on day 0 and 59.76 ± 1.23 on the 15th day of storage, while a trivial increase was recorded on the 30th day of storage. However, a gradual decrease was observed on the 45th and 60th days of storage at 58.96 ± 1.18 and 58.06 ± 1.44, respectively. Among the treatments, the minimum decrease was observed in T_0_ ranging from 95.90 ± 1.68 to 94.61 ± 0.67 from initiation to the termination of the research trial during storage. A maximum decrease was recorded in T2 (i.e., 45.08 ± 1.07 on day 0, 44.32 ± 1.06 on day 15, and 42.23 ± 1.98 on day 60 of storage), while a gradual decrease in L* value was observed in T_1_ and T_3_ as 53.06 ± 1.08 to 51.76 ± 1.54 and 46.07 ± 0.97 to 43.63 ± 1.58 at initiation and termination of the research trial during storage, respectively. The findings of the present study are in general agreement with those of Patras et al. (2011) who investigated the effect of storage on anthocyanins, ascorbic acid, total phenolic contents, and color of strawberry jam. During cold storage of 28 days, there was observed a decrease in L* values ranging from 18.3 ± 0.41 to 17.0 ± 0.12. They also concluded that during cold storage, a significant decline was observed in ascorbic acid and anthocyanin contents of strawberry jam. Kaur et al. (2011) studied the effect of addition of lycopene in ice cream to enhance the shelf life. During storage, there occurred a decline in the L value of control and treated samples ranging from 73.76 ± 0.14 to 72.60 ± 0.20 and 84.41 ± 0.31 to 83.97 ± 0.48, respectively. The current findings are also in agreement with the preceding work of Mazur et al. (2014) who verified the decreasing trend in L values of raspberry jam during storage.

#### 3.10.2 a* value

Means regarding the trait in microencapsulated black carrot anthocyanins powder‐enriched ice creams depicted a significant increase in a* values, which occurred in all treatments. The maximum value was observed in T_3_ (9% microencapsulated anthocyanins powder‐enriched ice cream) as 32.19 ± 1.2, followed by T_2_ (6% microencapsulated anthocyanins powder‐enriched ice cream) and T_1_ (3% microencapsulated anthocyanins powder‐enriched ice cream) as 29.25 ± 1.2 and 28.19 ± 1.08, respectively. However, the lower a* values were observed in T_0_ (control) (i.e., −1.97 ± 0.45). Over the storage, there were recorded significant differences in the mean values for this color parameter. Table 9 shows a clear decline in a* values as 22.85 ± 0.89 on day 0, followed by 22.40 ± 0.8 on the 15th day and 20.97 ± 0.98 on the 60th day of storage. Among the treatments, the same trend was observed and ranged between 29.21 ± 1.14 and 27.15 ± 1.08, 30.04 ± 0.96 and 28.31 ± 1.23, and 33.18 ± 1.04 and 31.19 ± 0.92 for T_1_, T_2_, and T_3_, respectively. The mean values for T_0_ at the beginning to the end of the research trial were −1.04 ± 0.44 to −2.78 ± 0.7, respectively. The earlier studies of Kaur et al. (2011) on addition of lycopene in ice cream to enhance its shelf life are matching to the findings of the instant research work, where there occurred a decline in a* values of the control and treated samples, ranging from 14.53 ± 0.33 to 13.21 ± 0.42 and 11.56 ± 0.09 to 11.16 ± 0.73, respectively. They also revealed that the use of natural colors derived from fruits and vegetables can reduce the risk of various diseases like cancer and atherosclerosis. The outcomes of the current work are also in corroboration with the research work of Patras et al. (2011) who investigated the effect of storage on anthocyanins, ascorbic acid, and total phenolic contents, along with the color of the strawberry jam. During cold storage for 28 days, there was observed a decrease in a* values ranging from 28.6 ± 0.31 to 20.5 ± 0.09. They also concluded that during cold storage, a significant decline was observed in ascorbic acid and anthocyanin contents of strawberry jam.

#### 3.10.3 b* value

During the study, a decrement in b* values was observed. The maximum b* values were observed in T_3_ (9% microencapsulated anthocyanins powder‐enriched ice cream) as evident from the mean (i.e., 10.31 ± 0.92). Among the treatments, the decrease in T_2_ (6% microencapsulated anthocyanins powder‐enriched ice cream) and T_0_ (control) was measured as 8.37 ± 1.04 and 6.51 ± 1.04, respectively. The minimum b * values were recorded in T_1_ (3% microencapsulated anthocyanins powder‐enriched ice cream) (i.e., 4.46 ± 1.26). During the storage, there was observed a steady decline in b* values as 8.11 ± 0.99 on day 0, followed by 7.80 ± 1.0 on 15th day, 7.04 ± 1.07 on 45th day, and 6.71 ± 1.20 on 60th day of storage. Among the treatments, a steady decline was recorded for T_0_ ranging from 7.16 ± 1.09 on day 0 to 5.90 ± 1.27 on day 60 of storage. Similarly, the b* values for T_1_, T_2_, and T_3_ were 5.14 ± 0.97 to 3.81 ± 1.50, 9.08 ± 0.95 to 7.67 ± 1.12, and 11.05 ± 0.96 to 9.46 ± 0.89 at initiation to termination of storage, respectively. The results of the current study are in concordance with those of Patras et al. (2011) who investigated the effect of storage on color, anthocyanins, ascorbic acid, and total phenolic contents of strawberry jam. During cold storage for 28 days, there was observed a decrease in b* values ranging from 8.7 ± 0.14 to 8.3 ± 0.85. They also concluded that during cold storage, a significant decline was observed in ascorbic acid and anthocyanin contents of strawberry jam. The earlier studies of Kaur et al. (2011) on the addition of lycopene in ice cream to enhance its shelf life are in line with the present findings, where there occurred a decline in b* values of control and treated samples ranging from 28.59 ± 0.17 to 26.79 ± 0.52 and 21.73 ± 0.23 to 21.29 ± 0.71, respectively.

#### 3.10.4 Chroma

Among the treatments, significant variations with respect to chroma were noted. It is inferred that a maximum value of chroma was observed in T_3_ (9% microencapsulated anthocyanins powder‐enriched ice cream) as 33.81 ± 1.24, followed by T_2_ (6% microencapsulated anthocyanins powder‐enriched ice cream) and T_1_ (3% microencapsulated anthocyanins powder‐enriched ice cream) as 30.44 ± 1.11 and 28.56 ± 1.11, respectively. However, the minimum value for chroma was observed in T_0_ (control) as 6.85 ± 1.06. Over the storage, there was observed a momentous decrease in chroma of ice cream which ranged between 25.82 ± 1.14 and 23.98 ± 1.10 from initiation to the termination of the research trial. The recorded values on the 15th and 45th days of storage were 25.42 ± 0.90 and 24.51 ± 1.20, respectively. Among the treatments, the maximum decline in chroma was observed in T3 ranging from 34.97 ± 1.29 on day 0 to 32.60 ± 0.81 on day 60 of storage. A steady decrease in chroma values was recorded in T_1_ and T_2_ as 29.67 ± 1.06 and 31.39 ± 1.07 at initiation to 27.44 ± 1.15 and 29.35 ± 1.04 on termination day. However, the minimum decrease in chroma was recorded in T_0_ as 7.24 ± 1.13 to 6.53 ± 1.40 from day 0 to day 60 of storage. The current findings are in general agreement with the preceding work of Patras et al. (2011) who verified the decreasing trend in chroma of strawberry jam during cold storage for 28 days. They also concluded that a significant decline was observed in ascorbic acid and anthocyanin contents of strawberry jam during cold storage. Mazur et al. (2014) also verified the decreasing trend in chroma of raspberry jam during storage for 6 months.

#### 3.10.5 Hue angle

It is illustrated that the treatments behaved varyingly with regard to hue angle. The maximum and minimum values were observed in T_0_ (control) and T_1_ (3% microencapsulated anthocyanins powder‐enriched ice cream) as 107.10 ± 3.46 and 8.95 ± 2.52, respectively. However, a clear demotion for hue angle was recorded in T_3_ (9% microencapsulated anthocyanins powder‐enriched ice cream) and T_2_ (6% microencapsulated anthocyanins powder‐enriched ice cream) as 17.74 ± 1.29 and 15.98 ± 2.14, respectively. Over the storage, there was observed a significant difference in the mean values for this attribute. Table 9 shows a slight increase in hue angle as 35.83 ± 1.82 on day 0, followed by 37.68 ± 2.35 on 30th day and 38.84 ± 2.72 on 60th day of storage. Among the treatments, a maximum difference in values was observed in T_0_ ranging between 98.09 ± 2.82z and 115.31 ± 3.57, respectively. However, the minimum values were recorded for T_3_ as 18.40 ± 0.97 to 16.89 ± 1.76 from initiation to the termination of the research trial, respectively. The findings of the current study are in accordance with the preceding work of Mazur et al. (2014) who investigated the effect of storage on color, ascorbic acid, and anthocyanins of raspberry jam. There occurred an 18% decline in hue of raspberry jam during 6 months of storage. They also reported that storage had a significant effect on anthocyanins content.

## 4 CONCLUSIONS

The present research demonstrated the potential of gum arabic and maltodextrin for the production of microencapsulated anthocyanins powder for further utilization in ice cream. The resultant encapsulates showed significant potential to stabilize the anthocyanins during processing and storage of ice cream. The microencapsulation strategy can further be exploited in other products as well to demonstrate its potential in different processing conditions. However, more vigorous research on encapsulated formulations may be devised to come up with more useful results in accordance with different product specifications. Also, such developed formulations used in products should be further explored for possible health outcomes.

## AUTHOR CONTRIBUTIONS

**Aneela Shamshad:** Conceptualization (equal); formal analysis (equal); methodology (equal); software (equal); writing – original draft (equal). **Iahtisham Ul Haq:** Formal analysis (equal); project administration (equal); supervision (equal); writing – original draft (equal); writing – review and editing (equal). **Masood Butt:** Investigation (equal); methodology (equal); project administration (equal); supervision (equal); writing – original draft (equal). **Gulzar Ahmad Nayik:** Conceptualization (equal); data curation (equal); methodology (equal); writing – review and editing (equal). **Sami Al Obaid:** Formal analysis (equal); funding acquisition (equal); investigation (equal); project administration (equal); resources (equal). **Mohammad Javed Ansari:** Methodology (equal); validation (equal); visualization (equal); writing – review and editing (equal). **Ioannis Konstantinos Karabagias:** Formal analysis (equal); investigation (equal); methodology (equal); writing – review and editing (equal). **Nazmul Sarwar:** Formal analysis (equal); resources (equal). **Seema Ramniwas:** Data curation (equal); formal analysis (equal); investigation (equal).

## CONFLICT OF INTEREST STATEMENT

The authors declare that they have no conflict of interest regarding the publication of this manuscript.

## Data Availability

The datasets generated during and/or analyzed during the current study are available from the corresponding author upon reasonable request.
